# Supplementary material for: Degradation of Proteoglycans and Collagen in Equine Meniscal Tissues
Source: Int J Mol Sci. 2024 Jun 11;25(12):6439. doi: 10.3390/ijms25126439 (PMC11203490; doi:10.3390/ijms25126439)
Supplement: Supplementary file 1 [file ijms-25-06439-s001.zip › ijms-2971320-supplementary.pdf]

International Journal of *Molecular Sciences*

MDPI

Supplementary Materials:

Table S1. Information on the archived stifles that were included in the current study. "The reason for euthanasia, when known, is indicated. All the collected joints were assessed for articular cartilage and meniscal changes as indicated in Tables S2 and S3"Key: QH = Quarterhorse; WB=Warmblood; F = female; G = gelding; M = male; R = Right; L = Left.

Horse identification	Age (years)	Breed	Sex	Stifle	Source	Reason for euthanasia
**1**	9	QH	F	L	Donation	Dental infection
**2**	10	Haflinger	F	R	Donation	Neurological signs
**3**	27	QH	G	L	Donation	Cachexia, respiratory disease
**4**	21	Appendix	G	L	Donation	Chronic intestinal disease
**5**	-	-	-	L	Abattoir	-
**6**	15	STD	F	L	Teaching herd	Intestinal disease
**7**	-	-	-	R	Abattoir	-
**8**	10	QH	G	L	Donation	-
**9**	-	-	-	L	Abattoir	
**10**	8	Pony	M	L	Donation	Chronic anorexia
**11**	3	Pony	M	R	Donation	Severe laminitis
**12**	26	QH	F	L	Donation	Age-related
**13**	23	STD	F	L	Teaching herd	-
**14**	23	QH	F	R	Donation	Age-related
**15**	-	-	-	R	Abattoir	-
**16**	11	Hanovarian	G	L	Donation	Severe stifle trauma
**17**	14	WB	G	L	Donation	Severe OA

CARTILAGE MACROSCOPIC SCORE	
0	Smooth and regular cartilage
1	Mildly irregular surface
2	Partial thickness erosion/fibrillation
3	Full thickness erosion

MENISCUS ROI MACROSCOPIC SCORE	
1	Normal intact meniscus<br>Sharp inner border<br>No surface change
2	No tears and either of the following<br>Fraying at inner border<br>Surface fibrillation
3	Partial substance tear<br>Fraying at inner border<br>Surface fibrillation
4	Full/Complete substance tears<br>Loss of tissue

Table S2. Criteria for the tissue bank stifle joint and meniscus macroscopic assessment scoring.

Macroscopic articular cartilage degeneration: The articular surfaces in the stifle joint were examined for cartilage changes, following the application of India ink, prior to inclusion in the bank These included the distal femur (medial and lateral femoral condyles and trochlear ridges,) and proximal tibia (medial and lateral tibial plateau) and patella (proximal, middle and distal) as described previously (1)The worst lesion of each area was scored employing a score adapted from a prior study (2). Data for the medial and lateral femorotibial compartments was retrieved from the archive for the present study to pair findings to the studied menisci.

Meniscal macroscopic degeneration: The macroscopic changes (fibrillation and tears) in each of the 3 regions (cranial horn; body and caudal horn), of either the tibial and femoral meniscal surfaces were scored employing a score adapted from Pauli et al. (3) following the application of India ink (1). The meniscus macroscopic ROI score was the sum of the scores for the femoral and tibial surfaces."

49. Dubuc, J., Girard, C., Richard, H., De Lasalle, J., and Laverty, S., Equine meniscal degeneration is associated with medial femorotibial osteoarthritis. Equine Vet J, 2017Jan;50(1):133-140.

50. Tiraloche G, Girard C, Chouinard L, Sampalis J, Moquin L, Ionescu M, Reiner A, Poole AR, Laverty S. Effect of oral glucosamine on cartilage degradation in a rabbit model of osteoarthritis. Arthritis Rheum. 2005 Apr;52(4):1118-28

51. Pauli C, Grogan SP, Patil S, Otsuki S, Hasegawa A, Koziol J, Lotz MK, D'Lima DD. Macroscopic and histopathologic analysis of human knee menisci in aging and osteoarthritis. Osteoarthritis Cartilage. 2011 Sep;19(9):1132-41.

ROI Identification	Meniscus	ROI Region	ROI meniscal macroscopic score	Femorotibial Compartment (L or M) OA score
1	L	Cr	3	1
2	L	Ca	4	1
3	L	Ca	3	1
4	L	Cr	2	0
5	L	B	2	0
6	M	Cr	2	4
7	L	B	2	1
8	L	Ca	4	1
9	L	Cr	4	2
10	L	Ca	2	2
11	L	Cr	2	2
12	L	B	4	2
13	M	Ca	2	4
14	L	Cr	2	1
15	L	B	2	2
16	M	Cr	4	5
17	M	Cr	6	6
18	M	B	4	6
19	M	Ca	4	6
20	L	B	4	1
21	M	Cr	4	3
22	L	B	3	1
23	L	Cr	4	1
24	M	B	4	5
25	L	Ca	3	0
26	M	Cr	4	1
27	M	B	4	3
28	L	Ca	5	6
29	M	B	4	5
30	M	B	4	1
31	M	Ca	4	1
32	L	Ca	4	4
33	M	Ca	5	6
34	L	Cr	3	2
35	M	Cr	4	9
36	M	Ca	3	1
37	M	Ca	4	8
38	L	Ca	6	3

39	M	Cr	4	3
40	M	Ca	4	3
41	L	Ca	2	0
42	M	B	5	6

Table S3. Information on the included ROIs and meniscus of origin.

Details on the origin of the meniscal ROI tissue provided. This includes medial (M) or lateral (L), the macroscopic score for the ROI prior to processing, and the femorotibial joint compartment cartilage degradation score of the joint of origin. The femorotibial joint compartment cartilage degradation score was extracted from the tissue bank archives and included the recorded scores from the Medial or lateral femoral condyles and corresponding tibial plateau surface and were summed to yield a compartment score for each meniscus.

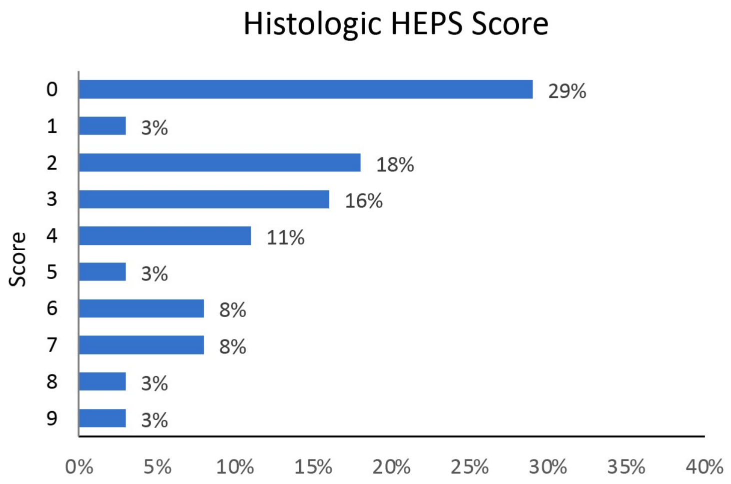

Table S4. The score frequencies of each of the histological scores in the ROIs.
